# Revealing the information contents of memory within the stimulus information representation framework

**DOI:** 10.1098/rstb.2019.0705

**Published:** 2020-04-06

**Authors:** Philippe G. Schyns, Jiayu Zhan, Rachael E. Jack, Robin A. A. Ince

**Affiliations:** 1 Institute of Neuroscience and Psychology, University of Glasgow, Scotland G12 8QB, UK; 2 School of Psychology, University of Glasgow, Scotland G12 8QB, UK

**Keywords:** memory, information processing, categorization, modelling

## Abstract

The information contents of memory are the cornerstone of the most influential models in cognition. To illustrate, consider that in predictive coding, a prediction implies that specific information is propagated down from memory through the visual hierarchy. Likewise, recognizing the input implies that sequentially accrued sensory evidence is successfully matched with memorized information (categorical knowledge). Although the existing models of prediction, memory, sensory representation and categorical decision are all implicitly cast within an information processing framework, it remains a challenge to precisely specify *what* this information is, and therefore *where*, *when* and *how* the architecture of the brain dynamically processes it to produce behaviour. Here, we review a framework that addresses these challenges for the studies of perception and categorization–stimulus information representation (SIR). We illustrate how SIR can reverse engineer the information contents of memory from behavioural and brain measures in the context of specific cognitive tasks that involve memory. We discuss two specific lessons from this approach that generally apply to memory studies: the importance of task, to constrain what the brain does, and of stimulus variations, to identify the specific information contents that are memorized, predicted, recalled and replayed.

This article is part of the Theo Murphy meeting issue ‘Memory reactivation: replaying events past, present and future’.

## 1. Introduction

**Figure 1.**
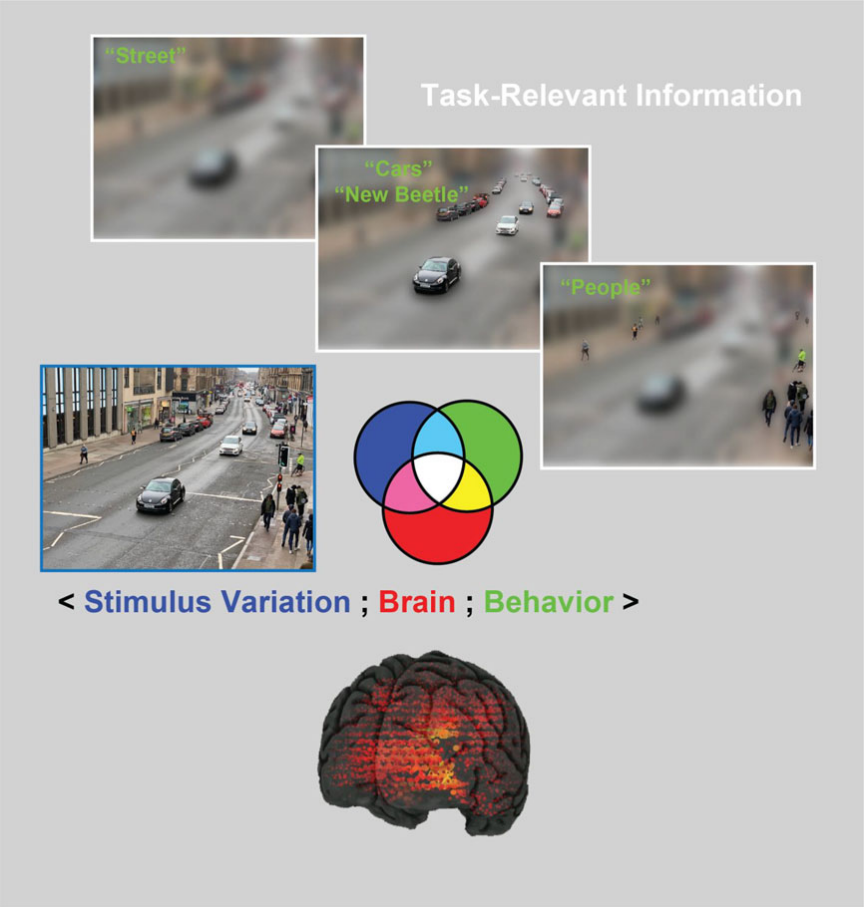
The human brain typically performs multiple categorization tasks on a single image using task-relevant stimulus information. For example, the brain uses coarse, global scene information to categorize this image as ‘street’ in a categorization task. By contrast, local details (i.e. other task-relevant information), as represented by finer image resolution, support other categorization tasks, such as ‘cars and their make/model’ and ‘people’. As we will illustrate, in the stimulus information representation (SIR) framework, samples of the stimulus are randomly generated and shown to participants to categorize (blue set). This approach generates variations in the participant's categorization behaviour (green set, e.g. ‘people’). Concurrently, participants' brain activity is recorded using neuroimaging techniques (such as EEG and MEG, see the red set) while they perform the task. The three-way interaction between these three SIR components (〈stimulus variation; brain; behaviour〉), as represented by the colour-coded set intersection, enables us to better understand *where*, *when* and *how* task-relevant information is processed in the brain.

The machinery that performs visual cognition has an extraordinary range of capabilities that is supported by the most powerful of sensory systems. Starting with the high-dimensional retinal input that comprises 150 M rods and cones, a large proportion of the cortex (30% to 60%) is then dedicated to accomplishing the feats of visual cognition. Among these is the ability to flexibly reduce the high-dimensional, highly variable input to flexibly categorize it to produce adaptive behaviours [1–3]. To illustrate, consider the street scene shown on the left-hand side of figure 1. The brain can perform numerous categorization tasks on this image: it can identify the country, city and street; the houses and shops; the moving and stationary cars, their makes and models, age and condition; the people shopping or those just passing by, as well as their gait, identity, emotion and social interactions; it can also infer the weather, time of day, season and so on. These are only very few of many categorizations that our brains perform apparently continuously and effortlessly.

To accomplish this feat, current models in the cognitive sciences assume that the categorization processes performed by the brain use memory representations, both to predict the incoming stimulus (e.g. a street, the type of a car or the emotion of a face) and also to categorize it (e.g. as ‘Byres Road’, ‘new Beetle’, ‘face’ or ‘happy’, figure 1). In these processes, the information contents stored in memory play a critical role in transforming the stimulus information that projects on the high-dimensional retina into categorization behaviour. However, it is notoriously difficult to understand what these memorized contents are, specifically. And without these contents, it is impossible to develop and test the information processing models of brain activity, the stumbling block of the neuroimaging agenda.

To bridge the interpretation gap, we developed the *stimulus information representation* (SIR) framework [4]. Specifically, we show that SIR can characterize the stimulus information contents stored in memory that are used to flexibly categorize incoming stimuli, and in turn to reveal *where*, *when* and *how* these contents are represented and processed in the brain. SIR comprises three critical elements that enable this interpretative step change: (i) rich stimulus variations with precise control, (ii) an explicit control of behaviour via specific cognitive tasks and (iii) a new analytical methodology that better decomposes brain activity (e.g. into that which represents task-relevant, versus task-irrelevant features). Though our applications of SIR are drawn from vision, the approach, which is based on the framework of psychophysics, is straightforwardly generalizable to any sensory modality.

Analyses of visual categorization processes with SIR provide a useful test bed to discuss the reactivation of neural activity that represents specific information during memory recall. For example, car experts would memorize the characteristic shape of the ‘new Beetle’ in figure 1. More casual observers could instead memorize general information about the car, leading to the same car being represented with different features in memory. Consequently, experts and novices could generally recall or replay different features about the same pictures. The key points here are first that observers can only predict or reactivate the information contents that they have memorized about a stimulus category [5–8] and second that we can generally expect these contents to vary to some degree across observers owing to relative category expertise. Hence, if we aim to understand the mechanisms that use memorized information contents (e.g. for categorization, as we will illustrate, but also for prediction and reactivation, as we will discuss), a useful starting point is to characterize *what* these contents are, to be able to trace their specific reactivation into brain activity, and thereby bridge the interpretation gap.

## 2. The stimulus information representation framework

The SIR framework starts with a critical constraint––a cognitive task––to limit what the brain is doing when we come to analyse its activity. As illustrated earlier in figure 1, a single stimulus can in principle recruit as many brain representations and processes as there are possible categorizations. However, typical brain-imaging studies of visual categorization are designed as if the stimulus (or a category of stimuli, e.g. *face*, *car* or *city*) on its own is sufficient to generate and therefore control specific representations and processes of the brain [9–11]. However, as illustrated, to achieve this necessary level of control, we must constrain the explicit cognitive task that the brain performs on the stimulus [12–15]. Next, having circumscribed a task, we then need to characterize the specific stimulus information that supports this categorization in memory [13,16]. Otherwise, we will not know what information the brain has used to make different categorizations such as ‘Byres road’, or ‘New Beetle’, ‘Face’ or ‘Happy’, from the same stimulus (figure 1).

Such task-relevant information processing is a generic, but often neglected, theoretical point that applies both to the interpretation of any sensory categorization in the brain and to its models. In the case of studies of memory, the tasks performed on a stimulus, or a stimulus category, even implicitly, impact the stimulus features that are memorized, particularly so when the input is high dimensional [5,7,17] (as in figure 1). In such situations, only partial, task-dependent stimulus representations are learned, and so when this stimulus is recalled, the current categorization task is likely to influence the stimulus features that will be recalled [5].

The SIR framework has been used to address such task-relevant information processing questions because it can isolate the specific stimulus information that drives variations in the brain activity that in turn generate variations in behavioural responses in a circumscribed task. For example, a typical design in the sciences of cognition involves multiple repeated trials of a specific task. SIR considers the three-way interactions between concurrent trial-by-trial variations of the three main components of such an experiment: stimulus variation, brain activity and behavioural responses. We will now detail each component and the procedure using concrete examples from vision science.

### (a) Stimulus variation

As we will illustrate with concrete examples, stimulus variation is crucial to explore, in a data-driven manner, a richer palette of brain and behavioural responses, as illustrated in figure 1. Stimulus variation can take different forms: it can consist of images generated by the random selection of pixels [16,18–20], or by sampling from the generative models of complex stimuli [21–25]. Such stimuli (as described in more detail below with images) are then presented to participants, who are asked to categorize them. So, in this first step, we randomly sample the variables that control stimulus information on each trial (as represented by the blue set in figure 1).

### (b) Behavioural measures

The second component of SIR consists of measuring behavioural variables in the performance of a categorization task such as response accuracy, reaction time or confidence ratings, on individual experimental trials (as represented by the green set in figure 1). Critically, when participants perform a categorization task on randomly generated stimuli, their behavioural responses can be used to disentangle what stimulus variables are relevant to that categorization task from those that are not. In this way, the participants' behavioural responses can reveal in a data-driven manner *what* information the participant (and therefore their brain) selectively uses to categorize information as ‘Byres road’, or ‘New Beetle’, ‘Face’ or ‘Happy’ (figure 1). Simply stated, we know the *what* of the information processing task.

### (c) Brain measures

Next, to understand *where*, *when* and *how* the brain processes the *what* (i.e. the task-relevant variables), we also measure its activity while participants perform the categorization task using various brain-imaging techniques, such as electroencephalography (EEG), magnetoencephalogram (MEG), functional magnetic resonance imaging (fMRI), near infrared spectroscopy (NIRS), electrocorticography (ECoG) or single-cell recordings. The red-shaded set in figure 1 represents variables in brain activity recorded during a task, as recorded by different sensors, at different sources or time points, or three-dimensional voxels of bold activity, or representing different neuron firing rates.

Together, the three components of SIR test how randomly sampled stimulus information influences brain activity and behaviour during a particular categorization task. In addition, the three-way interactions among these three components, as denoted by 〈stimulus information; brain; behaviour〉, are represented as the four-colour-coded set of intersections shown in figure 1 (that is, the blue, green and red sets of the Venn-like diagram, and the white, light blue, magenta and yellow areas where they intersect). With these three-way interactions, we can then model the information contents of the brain with unmatched interpretative precision, as we illustrate in the following sections.

## 3. Applying stimulus information representation to model and understand task-relevant information

Here, we illustrate how SIR brings these components together to model, using reverse engineering, the processing of stimulus information in the brain while it is performing a categorization task. We first show the two-way interactions before extending to the three-way interactions. In the first three examples, we reverse engineer the information contents of the face memory of individual participants (i.e. the *what*) from the two-way interactions between stimulus variation and three different face categorization tasks (i.e. face detection, face identity and facial expressions of emotion across cultures). In the final example, we use the three-way interactions (as represented by the white triple set intersection) to reverse engineer *where*, *when* and *how* the brain dynamically processes task-relevant (and task-irrelevant) information in a perceptual decision task. We now review these components of the SIR framework in turn.

### (a) Face detection

**Figure 2.**
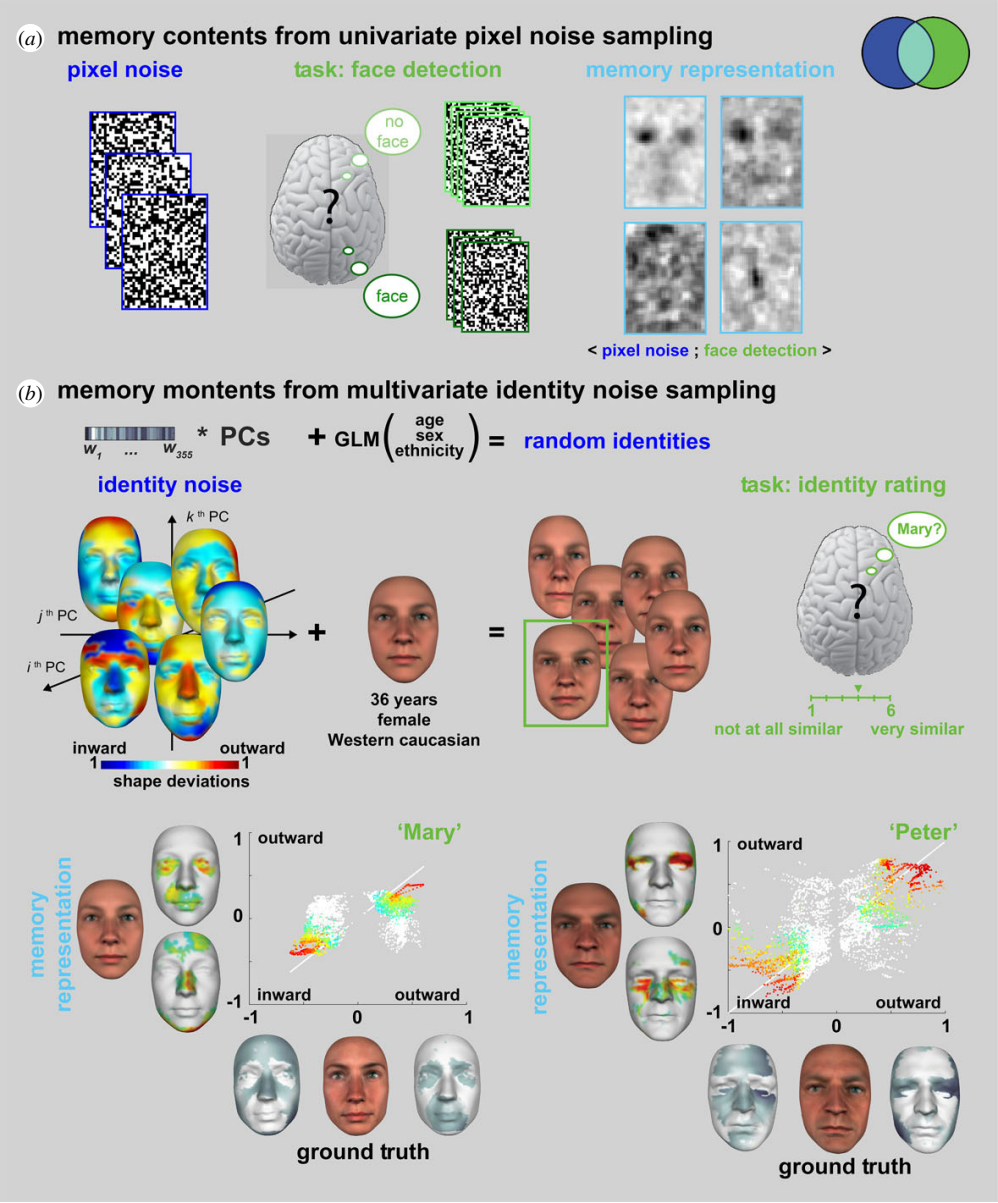
Reverse engineering the contents of face memory (adapted from [13,26]). (*a)* Memory contents from univariate pixel sampling [13,19]*.* Individual participants only saw pixel noise on each trial but were instructed that a face was hidden in the noise on half of the trials. They were tasked to detect the face by responding ‘face’ versus ‘no face’ on each trial. Following the experiment, we computed the classification image (i.e. the relationship 〈Pixel Noise; Face Detection〉) to reverse engineer the information contents of the memory prediction for a face. (*b*) Memory contents from multivariate identity noise sampling [26]*.* Individual participants saw six random identities controlled on each trial by a generative model of face information. This generative model involves local averages based on gender, age and ethnicity, to which unique random residuals are added to generate random identities. In this experiment, randomly sampled identities were added to the same gender, age and ethnicity as the target face (e.g. ‘Mary’, a Western Caucasian female of 36 years of age). On each trial, participants were tasked to choose the random face that looks most like ‘Mary’ and to rate this similarity on a 1 to 6 Likert scale. Following the experiment, we computed the relationship 〈Identity Noise; Similarity to ‘Mary’〉 to reverse engineer the information contents of familiar face memory. We then compared the memory representations of ‘Mary’ (left panel) and ‘Peter’ (right panel) with the objective ground truth information that defines these familiar identities in the face model. Coloured vertices indicate the vertices that faithfully represent ‘Mary’ in memory. Vertex colours in the scatter plots are reported as colour-coded vertices in the memory representations.

Suppose we instruct each participant that they will see white noise images (figure 2*a*) and that half of them comprise a face well hidden in the noise (or, in another experiment, the letter ‘S’ hidden in noise). The trick here is that there is never a face (nor a letter) in the noise stimuli; participants are only presented with white noise. To resolve the task, each participant will use memorized information to *predict* what a face (or letter) should look like and then match this prediction with the incoming input—i.e. pixel noise presented on each trial.

A small positive correlation between the prediction from memory and the noise drives the observer to respond ‘face’ (or ‘S’), even when the input is only white noise. We can reverse engineer the memory prediction that guides such decisions by correlating each individual pixel (which is either black or white on each trial) with the corresponding detection response of the participant (i.e. ‘face’ versus ‘noise’ on each trial, or ‘S’ versus ‘noise’ in the other experiment). The resulting *classification image* (figure 2*a*) models the information contents of the memory prediction for a face (or ‘S’ letter) in each participant [13,18–20]. In SIR, the classification image is the light blue intersection that comprises the randomly sampled pixel variables (in the blue set) that are relevant for task behaviour (in the green set).

A foundational justification for sampling white noise to reverse engineer the information contents of memory is based on the Volterra and Wiener system identification in engineering. It states that we can identify a complex system from a linear sum of subsystems *F_i_* by estimating the kernels *f_i_* of each subsystem *F_i_*, where the kernels are the orders of representation of information in memory [20,27–31]. For example, the light blue intersection in figure 2*a* is the first-order (i.e. pixel-by-pixel, univariate) kernel f_1_ captured by computing the relationship 〈white noise pixel; detection behaviour〉. Higher-order kernels can be added to this first-order memory estimate, by computing 〈white noise pixel*^n^*; detection behaviour〉, for each order *n* of nonlinear relation between the sampled pixels—i.e. the f_2_ kernel of pairwise pixel relations, the f_3_ kernel of triplewise relations and generally the f*_n_* kernel of *n*-wise pixel relations. This approach has fruitfully modelled the way neurons process specific, low-dimensional sensory attributes in visual [32], auditory [33,34], memory [35] and somatosensory systems [36], see [31] for a review.

A key advantage of such white noise sampling is the few assumptions that it makes about the structure of information in memory, letting instead a data-driven computation based on behavioural responses to discover this structure as the *f_i_* kernels of the Volterra–Wiener expansion. However, this approach is impractical with images of realistic faces, objects and scenes, where each individual pixel becomes a first-order parameter to estimate, together with the many orders of interactions between individual pixels that make up natural image features. Furthermore, natural image features form densely correlated clusters of pixels, whose occurrence will be extremely rare if each image pixel is independently and randomly sampled. Thus, the finite time of a human experiment will make it practically near impossible to estimate the memorized representations of natural image features using white noise pixel stimuli. To address these shortcomings, a fruitful approach is to hypothesize that memorized information is structured and then embed the structuring hypotheses as the explicit dimensions of a generative model of the stimulus. The next examples apply this generative approach in two different tasks.

### (b) Face identity

Here, the task is to identify a familiar face from memory [26]. What stimulus information should we randomly generate to reverse engineer the memorized information that guides this task? As discussed, a starting point is to hypothesize, and then model, the information structure of real-world human faces. We note that human faces are statistically smooth, textured surfaces that systematically vary according to their age, sex, ethnicity and, of course, identity. A good model would therefore isolate the information that uniquely identifies a face from that which is shared across faces to represent their age, sex and ethnicity. We formalized these constraints within a linear model that separates the identity component of a face (e.g. ‘Mary’) from the component that represents their shared age, sex and ethnicity as a local average (e.g. the average of all 36-year-old Western white females).

Equipped with this explicit model, we come back to the original question of the identity noise that we should generate to reverse engineer the contents of familiar face memory. Suppose that the target face is ‘Mary’, a 36-year-old Western white female well known to her colleagues. To generate identity noise, we use the two-component model just discussed, and within it set the local average to represent all 36-year-old Western white females. We add to this shared average a randomly generated component of identity as illustrated with the six random identities in figure 2*b*. Critically, these faces share the same local average age, sex and ethnicity as ‘Mary’ and only differ in identity. On each trial, we then ask the participant to select the face (among the six presented) most similar to ‘Mary’ and to rate this similarity on a 1–6 scale from ‘not at all similar’ to ‘very similar’. Following the experiment, we compute for each participant the light blue intersection 〈identity noise; similarity to ‘Mary’〉 for each multivariate component that controls the shape identity noise (and texture identity noise, not shown). The result models the memory contents of a familiar face.

For each participant, we can then compare these memory contents with the ground truth—i.e. the objective identity information of the familiar face. To illustrate, grey faces on the *x*-axis of figure 2*b* show the ground truth identity that defines ‘Mary’ (and ‘Peter’) as Inward and Outward three-dimensional shape deviations in relation to the shared local average. For example, Mary's nose is objectively thinner than average and so these vertices deviate inward (darker grey tones indicate increasing deviations). Likewise, her more pouty mouth is shown as an outward three-dimensional shape deviation. The *y*-axis of figure 2*a* uses the same format to show the memory contents for ‘Mary’ in one typical participant, where colours indicate increasing deviations. These contents reveal faithful representations of, for example, a thinner nose and a pouty mouth. The scatter plot visualizes the vertex by vertex fit between the memory representation (*y*-axis) and the ground truth three-dimensional face (*x*-axis). The white diagonal line provides a veridical reference, where the identity component in the memory representation is identical to the ground truth face, for every single three-dimensional vertex.

There is an important difference between the white noise used in the face detection example and the component identity noise used here. Whereas the white noise was pixel-by-pixel (i.e. 32 × 43 = 1376 univariate parameters), making few assumptions about the structure of memory representations, the multivariate identity noise (with 355 components of shape and 355 components of textures = 710 parameters) is derived from a generative model of real face information that is laden with explicit hypotheses (i.e. linearity and independence of identity from the age, sex and ethnicity factors). Of course, both types of noise enabled the reverse engineering of task-relevant memory contents, at the level of individual participants and with greater precision and fewer parameters (and therefore trials to estimate them) with the multivariate identity noise. However, a sceptic would correctly point out that these more precise reconstructions are essentially model bound and that we are compromising the open-endedness of a data-driven approach with each structural hypothesis—i.e. we can only reconstruct the memory information in relation to the structural hypotheses explicitly formulated in the generative model. This is indeed an important point that requires careful consideration.

### (c) Facial expressions of emotion

**Figure 3.**
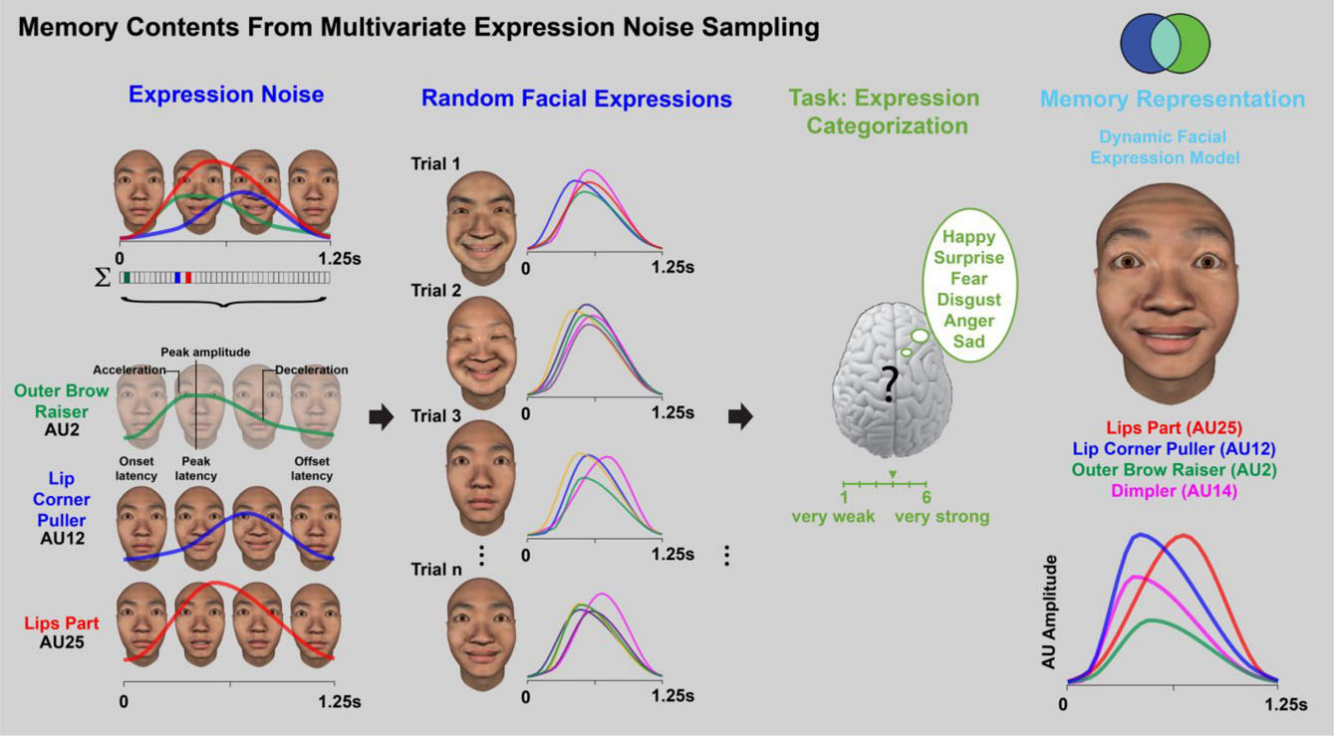
Reverse engineering the contents of facial expression memory (adapted from [23]). On each trial, individual face movements and their 7 dynamic parameters are randomly sampled to produce a 1.25 s facial animation (see coloured curves). Participants from two cultures are asked to categorize the animation by emotion (6 classic emotions plus ‘other’) and intensity (on a 5-point Likert scale from very weak to very strong). Across trials, we model the memory representation of each facial expression by computing the individual face movements (and their dynamic parameters) that are systematically associated with each emotion category response and intensity level.

In this third example [37], we turn to the dynamic memory information that guides the categorization of facial expressions of the six classic emotions (i.e. ‘happy’, ‘surprise’, ‘fear’, ‘anger’, ‘disgust’ and ‘sad’) in two cultures (i.e. white and East Asian). Here, we will again sample a multivariate noise to reverse engineer the contents of facial expression memory. However, as real-world facial expressions involve specific facial movements called action units (AUs), we incorporate AUs and their dynamic parameters as structural hypotheses of a generative model of random AU movements, as illustrated in figure 3. On each experimental trial, Western Caucasian (WC) and East Asian (EA) participants viewed a 1.25 s facial animation that randomly sampled and combined a subset of multivariate AUs from a core set of 42 AUs. For example, in figure 3, three AUs are selected: outer brow raiser (AU2) colour-coded in green, lip corner puller (AU12) in blue and lips part (AU25) in red. Each is activated with a random movement (see colour-coded temporal activation curves for each AU; temporal parameters are labelled in the green curve).

Participants viewed the facial animation, classified it according to one of the six emotions and rated its intensity on a 6-point scale (from ‘very weak’ to ‘very strong’). If the facial animation did not correspond to any of these emotions, participants selected ‘other.’ After many such trials, we reverse engineered the memorized information for each facial expression by building the light blue statistical relationship 〈AUs; expression category〉 between the dynamic AUs presented on each trial (the blue set of stimulus variables in the blue set) and the participant's corresponding responses (e.g. ‘happy’ figure 3, the green set of response variables). The memory models revealed a culture-specific representation of the temporal dynamics of emotional intensity. Specifically, whereas EA participants represent emotional intensity primarily with early movements of the eyes, WC participants represent emotional intensity primarily with the mouth [37] (see electronic supplementary material, Video S1). This divergence is mirrored in the emoticons of popular culture, where ‘happy’ is represented with ‘^ ^’ in the EA culture and with ‘:)’ in the WC culture.

## 4. Applying stimulus information representation to model and understand task information processing in the brain

So far, the examples have focused on reverse engineering task-relevant information, the light blue, two-way interaction of the SIR framework (represented as the light blue intersection between the blue set of pixel samples and the green set of corresponding behavioural responses). Such task-relevant features, represented as univariate features or multivariate shape of expressive components, are pivotal for understanding information processing in the brain because they represent the stimulus features that the brain must process to accomplish the behavioural task in question. In other words, task-relevant features are the needles of information that we should search for in the haystack of brain activity. To find these features, we now intersect the third component of SIR––in this last example, MEG signals, recorded on each trial in response to random stimulus samples that are behaviourally categorized. We can now explore the richer three-way interactions between stimulus variation, brain measures and behaviour (as 〈stimulus variation; brain; behaviour〉).

**Figure 4.**
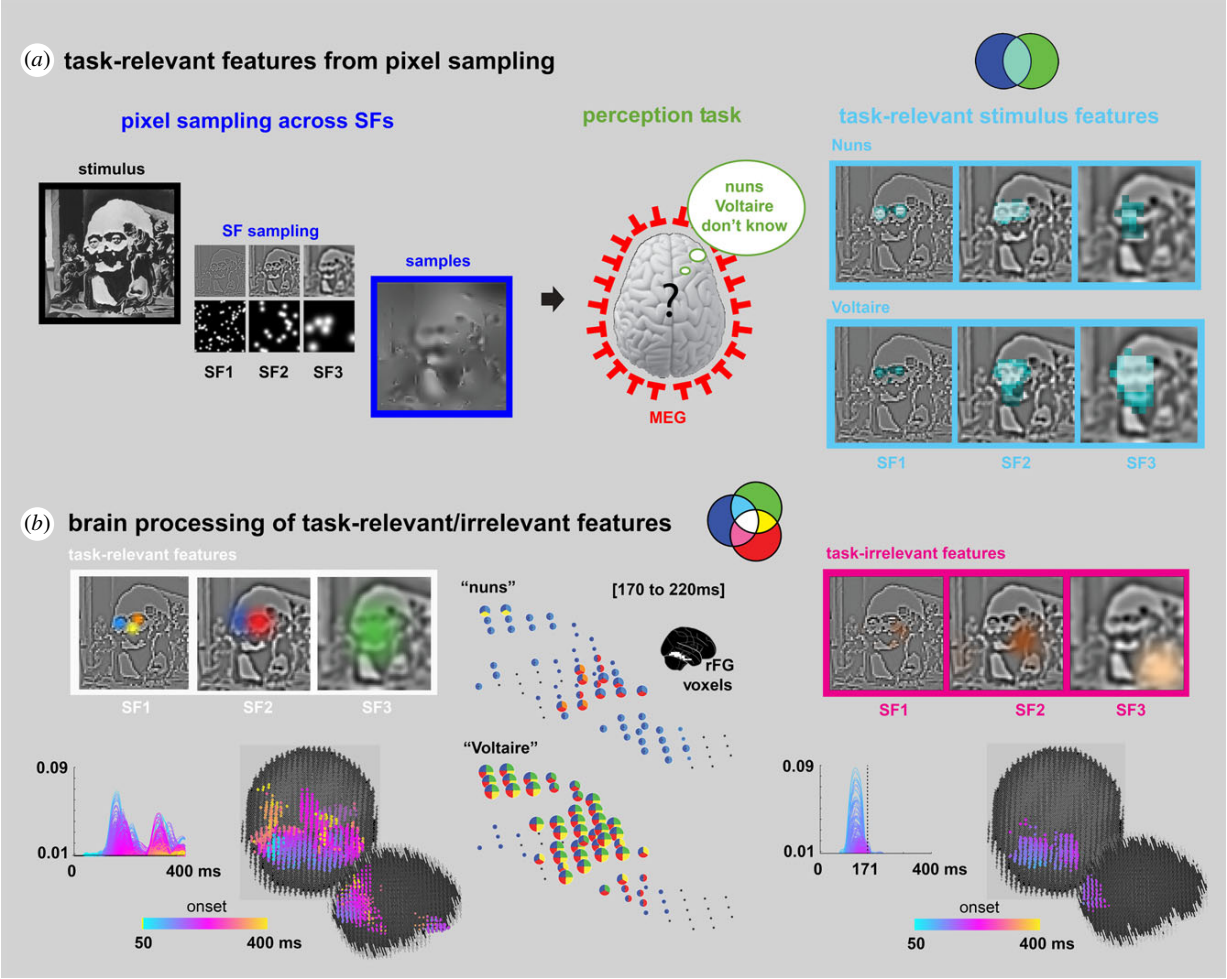
Using SIR to study the processing of detailed information contents in the brain (adapted from [4]). (*a*) Task-relevant features from pixel sampling*.* Random sampling of pixels across spatial frequency (SF) bands of an ambiguous stimulus using Bubbles [16]. Participants viewed the resulting sparse images (framed in blue) and were tasked to categorize each as ‘Nuns’, ‘Voltaire’ or ‘don't know’, while we recorded their brain activity (via MEG) and decision behaviour. The pairwise relationship 〈Pixel; Decision〉 for each image pixel was computed to reveal task-relevant (light blue-shaded) features of each decision. (*b*) Brain processing of task-relevant/irrelevant features. Three-way relationships were also computed to reveal task-relevant feature representation (the white triple intersection, white framed features) and task-irrelevant feature representation (the magenta set remainder of magenta framed features) in brain activity. With SIR we showed *where*, *when* and *how* specific task-relevant features combine in the right Fusiform gyrus (rFG) into decision-specific representations. Pie charts standing in for rFG voxels indicate the representational strength (computed from the triple relationship 〈Pixel; MEG; Decision〉) of the colour-coded task-relevant features framed in white in panel. To illustrate, the blue and orange features respectively representing the left and right nuns faces in SF1 are selectively represented in rFG voxel activity for ‘the nuns’ response, whereas the green feature representing the face of Voltaire in SF3 is broadly represented across rFG voxel activity for ‘Voltaire’ responses. The colour-coded plots and corresponding brains at the bottom indicate the strength of representation of task-relevant (left panel) and task-irrelevant (right panel) features. Task-irrelevant features are initially represented and then dynamically filtered out in occipital cortex approximately 170 ms post-stimulus.

The following illustrates how this triple intersection produces the coloured intersections of SIR that can be used to disentangle brain activity into the processing of task-relevant stimulus information, the processing of task-irrelevant stimulus information and other brain processes, to enrich the interpretation of brain-imaging data. Figure 4 illustrates the task [38,39], where each participant saw an ambiguous image (stimulus) that can be perceived as being either ‘the nuns’, or ‘Voltaire’ (squint to see the latter, figure 4). In this task, the green set consists of the participants' response variable across trials, which can either be ‘the nuns,’ or ‘Voltaire,’ or ‘don't know.’ As before, to understand the task-relevant information for each of these responses, we systematically and randomly sampled the image to reveal different pixels to a participant on each trial. The blue set shown in figure 4 thus includes each image pixel as a distinct stimulus variable (with ‘on’ or ‘off’ values), based on random sampling across trials.

Using the blue set of randomly sampled image pixels and the green set of corresponding behavioural responses, we then infer the task-relevant stimulus features for each perception by computing 〈pixel; behavioural decision〉, using (mass-bivariate) pairwise relationships. This computation disentangles all image pixels into those that are relevant for task behaviour (i.e. for participants to categorize an image as being ‘the nuns’ or ‘Voltaire’, as represented by the light blue intersection shown in figure 4) from those that are not (which are encompassed in the remainder of the blue set), just as before.

To find where, when and how the brain processes these light blue, task-relevant pixels we intersect the third component of SIR: MEG signals, which were recorded during the task. We then compute the overlapping co-representation of the stimulus features into the behaviour and brain measures (as 〈pixel; MEG; decision〉). The outcome of this computation identifies the light blue task-relevant features that the red set of MEG variables represent, as highlighted by the white triple intersection of the three components of SIR.

Zhan *et al*. [4] used these three SIR components to trace the dynamic flow of task-relevant features that were processed between 50 and 220 ms post-stimulus, from their early representation in the visual cortex, through the ventral pathway. In the ventral pathway, they found that task-relevant features converge onto a few MEG voxels at the top of the right fusiform gyrus, approximately 200 ms post-stimulus, where they agglomerate into distinct representations that support each behavioural decision (see the colour-coded ‘task-relevant’ features in figure 4 and their corresponding colour-coded pie chart representations between 170 and 220 ms post-stimulus on the rFG voxels). Thus, using the three-way interaction between the components of SIR, Zhan *et al*. traced the dynamics of task-relevant feature processing over the first 220 ms post-stimulus, from their early representation in visual cortex to their integration for each behavioural decision in the ventral pathway.

## 5. What do we learn from the intersections of SIR?

To re-cap, in the example just reviewed, we have three concurrent datasets in the SIR framework: the blue set of stimulus information samples, the red set of brain measures and the green set of behavioural responses in a task, along with their four intersections (coloured white, light blue, magenta and yellow, as shown on figure 1). The white triple set intersection is transformative for neuroscience and neuroimaging because it disentangles the different relationships between stimulus, brain activity and behaviour as follows. The white set divides each coloured intersection into the white component and a remainder. Each of these four intersections contributes its own unique component of interpretation to provide a more detailed understanding of the processing of information contents in the brain. We review each intersection in turn.

### (a) The light blue intersection

Within a given task, the light blue remainder set isolates and represents the task-relevant features that the recorded brain measures do not represent. This remainder flags a *de facto* incomplete explanation of the processing of the stimulus information that supports a particular behaviour. This is because all task-relevant features should be represented somewhere in the brain to influence behaviour. A complete brain measure (such as that captured by a brain-imaging modality) should entirely absorb the light blue remainder (of task-relevant features) into the white set intersection (the light blue remainder is empty in the example shown in figure 4, where the white framed task-relevant features are all processed for behavioural decision in the brain).

### (b) The magenta and yellow intersections

A magenta remainder reveals task-irrelevant stimulus features, which the brain represents but which do not directly influence behaviour in the task. In figure 4, the information processes reduce (i.e. filter out) a travelling wavefront of task-irrelevant feature representations within occipital cortex, around 170 ms post-stimulus (compare the magenta ‘task-irrelevant’ features and brain in figure 4 to the white ‘task-relevant’ counterparts). Importantly, although these task-irrelevant features do not influence the participants' categorization responses, they were among the features that were most strongly represented in brain activity in early visual cortex. However, they do not reach the fusiform gyrus in the ventral pathway, as the task-relevant features do.

Finally, the yellow remainder isolates brain activity that relates to a behaviour but not to stimulus variation. These brain processes likely reflect other aspects of the task, such as modulation of arousal, response planning, response bias, execution and so forth.

## 6. General discussion

Here, we argued that the information contents of memory are the cornerstone of the most influential models of prediction, representation of categories in memory and categorical decision. Although these models are all implicitly cast within an information processing framework, the challenge is now to precisely specify *what* this information is, and *where*, *when* and *how* the architecture of the brain dynamically processes it to produce behaviour. We reviewed SIR, a framework that can address these challenges in individual participants while they are actively performing an explicit task. During such tasks, stimulus variations are applied on each trial to cause concurrent variations in the participant's brain activity and behaviour. Here, using three face tasks, we illustrated how SIR can reverse engineer, in a data-driven manner, the task-relevant information contents of memory by computing the two-way interactions between behaviour and the univariate, low-structured pixel noise (in face detection), or the multivariate, hypothesis-rich components of a generative model of face shape or movement (in face identity or emotion, respectively). Note that such designs could have been augmented by changing the participant's brain activity with a cue [40], with direct interaction using repetitive transcranial magnetic stimulation (TMS) [41–43], or other methods, thereby changing the information sampled and behaviour. We argued that task-relevant features are the needles of information that we should search for in the haystack of brain activity and computed the triple interactions of SIR to do so (represented as the coloured intersections). With these, we showed how we can trace the processing of task-relevant and task-irrelevant features in the brain between stimulus onset and behavioural decisions.

## 7. Using rich stimulus variations in a task to infer information contents at recall

Our applications of SIR identified the variety of stimulus features that can represent a visual category in the memory of individual participants. We also showed how we can track neural representations of these features into the variations of dynamic MEG activity (and also in other measures such as EEG [12–15], fMRI [44] and single-cell recordings [45]). Our analyses use the important interactions between the bottom-up random variations of stimulus components and their top-down usage guided by memorized knowledge. Using these we can separate out the representation of task-relevant from task-irrelevant features in the brain [4]. In SIR, an explicit generative model of the stimulus is necessary to produce the variations of stimulus features that can tap into multiple categorizations of the same stimulus (or category). These considerations also apply to identifying the memory contents supporting different categorizations of a single face (e.g. identity, emotion or social trait), or different levels of expertise of the same object category (cf. ‘new Beetle’ versus ‘car’ or ‘German Shepherd’ versus ‘dog’ or ‘New York’ versus ‘city’). As we will discuss, we can design hierarchical generative models to tap into taxonomies of categories in memory.

Turning to cued recall of information contents from memory, we can use the SIR framework to start addressing two broad questions. First, how can we establish what information a specific brain activity recorded during recall represents (whether EEG, MEG, fMRI or single unit recording) and how does this relate to the information represented during encoding? Second, what are the effects of the explicit behavioural task, both during encoding and at recall, on the information represented, and how do these interact? Though there is at present no methodology to address these questions directly, we refer the reader to figure 4 to start considering how the information contents derived using the SIR framework could inform information-rich studies of cued recall. In figure 4, we used a perceptual categorization task and computed the rich variety of stimulus features that each individual participant processed to enable the response ‘the nuns’ (i.e. the fine-grained left and right nuns' faces) and ‘Voltaire’ (i.e. the coarser face of Voltaire and his eyes). Let's now examine how we could use this analysis to inform the information contents of brain activity during recall.

We could compute the explicit MEG signature of each one of the stimulus features at encoding (as was done in [12,46]). We could then compare the neural signatures of the specific features of each participant with the activity elicited when the participant would recall each perception in the absence of an explicit stimulus, for example with a cross-decoding analysis (train classifier during encoding, test at recall) as has been successfully applied to fMRI [47,48], EEG/MEG [49–53] measures. The balance of represented features might change between encoding and recall depending on the task considered in each. Such an approach could establish a similarity of brain activity between stimulus encoding and recall and, via the analyses of SIR, indirectly inform the specific features that a participant activates when recalling ‘the nuns’ or ‘Voltaire.’ This thought experiment could generalize to studies of the information contents of other face, object and scene categorizations, between their encoding and recall.

Stepping back from the example, we make an explicit, functional theoretical point about the information contents of memory: the representation of any stimulus category should at least comprise the stimulus features that afforded its multiple categorizations in the history of the individual categorizer [5,7,8] (e.g. a variety of features to categorize the relevant identities, expressions, ages, ethnicities and social traits for faces, and the taxonomic categorizations of the objects and scenes at the required levels of expertise [54]). The SIR framework provides the analysis tools to identify *what* these features of the stimulus are in memory. Consequently, even recalling the same scene stimulus could elicit different neural activity if we tasked the participant with recalling whether the cued stimulus is a city, New York, or a view of the Chrysler building, because these categorizations would require different functional features, each with a specific neural signature.

We have focussed so far on cued recall, where the framework of SIR (including rich stimulus variation of multiple stimulus features and explicit consideration of task) has clear applicability. However, this approach could also give insights into information-bearing reactivations during offline replay. For example, a richer design including multiple stimulus features and different tasks could reveal that the specific feature representations reactivated during offline replay differ depending on the explicit task performed during learning. Here again, the SIR perspective could be combined with existing cross-decoding approaches, enhancing them with the key features of carefully designed rich stimulus variation and explicit control of the cognitive task. In the offline replay setting, there are no explicit behavioural responses, but we might still expect to see an effect of the categorization task during sequence learning on the specific nature of the offline replay.

## 8. Extension to events, situations and sequences

We now sketch how generative models could be extended to the object [21] and scene categories [22] making up events and situations. Consider designing the generative models of two city scenes built around one prototype plus a specific component that identifies each scene. At testing, the scene component would be randomized and discrimination performance measured. With reverse correlation, we could reverse engineer from behaviour the features that each participant represents in memory for each scene. We could then use the framework of SIR to compute the encoding models of each component and test whether we can elicit similar brain activations for prediction or recall, when faced with a blank screen. A similar approach could be applied to the design of articulated object categories [21]. However, the main drawback of such approaches is the large experimental time that such an experiment would require, unless the generative model was itself multivariate (e.g. different blocks of buildings to generate the two scene categories, rather than individual buildings) and we could develop encoding models of these multivariate components in the brain. These expansions are part of our on-going research. Our methodology is also in principle extensible to studies of memory that use structured sequences, where the specification of a task is also important. For example, the sequential replay of a memorized sequence of images similar to figure 1 might differ depending on whether the participant was asked to count the total number of people versus the total number of cars visible in the sequence.

## 9. Conclusion

Generalizing from our examples, using a range of modern imaging modalities, we can now measure on individual trials the brain activity of an individual participant while they are actively performing an explicit task. SIR takes full advantage of these richer datasets by computing the interactions between these three components, as 〈stimulus variation; brain; behaviour〉. These computations can then be used to disentangle the stimulus, brain activity and response spaces, including at different granularities, depending on the specific experimental design. From these, we make inferences about what information is being processed in the brain for a particular behaviour, and where and when this processing occurs, which in turn can inform computational models of behaviour. SIR can also be applied to study any parametrizable sensory stimulus spaces (e.g. auditory [24,25,34,55], as well as other cognitive, social and affective tasks; for reviews see [23,56,57]) and to study the information processing mechanisms of both brain and in silico architectures. We propose, therefore, that the time is ripe to exploit the full capabilities of modern brain-imaging technologies and to embrace richer designs that exploit the trial-by-trial trivariate 〈stimulus information; brain; behaviour〉. The analysis of such richer designs within the SIR framework may reveal novel interactions that further our understanding of how the brain processes information contents for behaviour.

SIR provides a number of benefits for studies of memory recall and replay. As we have seen, by focusing on explicit tasks performed on a remembered stimulus, or stimulus category, SIR can specify the information contents that must be recalled to facilitate behaviour and then guide the search for replay of that specific information in brain activity. As with categorization and visual perception, an increased focus on explicit task control and task-relevant information content is critically important to understand the information contents of memory.

## Supplementary Material

Culture-specific representations of the same emotions (i.e. happy, disgust and anger).

## Data accessibility

This article has no additional data.
